# No Decrease in Infection Rate with the Use of Local Vancomycin Powder After Partial Hip Replacement in Elderly Patients with Comorbidities

**DOI:** 10.7759/cureus.10296

**Published:** 2020-09-07

**Authors:** H. Yener Erken, Gurdal Nusran, Doğaç Karagüven, Onur Yilmaz, Tolgahan Kuru

**Affiliations:** 1 Orthopaedics and Traumatology, Canakkale Onsekiz Mart University, Canakkale, TUR; 2 Orthopaedics and Traumatology, Ufuk University, Ankara, TUR

**Keywords:** hip arthroplasty, local application, partial hip replacement, surgical site infection, vancomycin powder

## Abstract

Introduction

The goal of this study was to evaluate the effects of local intra-wound vancomycin powder (VP) administration to decrease surgical site infections (SSIs), particularly in elderly patients with comorbidities, after having undergone partial hip replacement in the treatment of intertrochanteric (ITF) or femoral neck fractures (FNF).

Methods

We retrospectively reviewed patients who underwent partial hip replacement in the treatment of ITF or FNF in one year. We divided the patients into two groups. The non vancomycin-treated group received standard systemic prophylaxis only (1 gr cefazolin IV), while the vancomycin-treated group received 1 gr of VP in the surgical wound just before surgical closure in addition to the systemic prophylaxis. We included patients of 64 years or older who also had one or more comorbidities. We compared the post-operative SSI rates between the non vancomycin-treated group and the vancomycin-treated group.

Results

A total of 93 patients were included in the study. We detected post-operative wound infection in six patients (6.4%). The rate of SSI was found to be 5.7% in the vancomycin-treated group and 6.9% in the non vancomycin-treated group respectively, which showed no statistically significant difference (p:0.498). The incidence of SSI was statistically higher in the patients who had a follow-up in the post-operative intensive care unit than the patients who had not any follow-up in the intensive care unit.

Conclusion

Local application of VP in the surgical wound was found to be ineffective in reducing the incidence of SSI after partial hip replacement in elderly patients with comorbidities.

## Introduction

Surgical site infections (SSIs) after hip replacement surgery cause high morbidity, mortality, and economic cost to the patient [1-3]. Therefore, various prophylactic methods are applied before, during, and after surgery to minimize the risk of post-operative SSIs. Treatment of infections after hip arthroplasty often adversely affects the quality of life of the patients and their families, as long-term antibiotic therapy, multiple surgical procedures, and long-term follow-ups are often required. In a rabbit spine-infection model, it has been shown that local administration of vancomycin powder (VP) reduces the rate of SSI [4]. In a recent study, O'Neill et al. showed that the use of VP in surgical wounds may significantly reduce the incidence of infection in patients with traumatic spine injuries treated with posterior spine fusion and instrumentation (PSFI) [5].

Efficacy and safety of intrawound VP application has been shown to decrease post-operative spine infections, but its use in arthroplasty has not been well established. There are limited number of studies which assess the effect of intrawound VP administration to reduce SSIs after hip arthroplasty. However, none of these studies were specifically performed on elderly patients with comorbidities who are already more prone to post-operative SSIs than younger, healthier patients. In the present study, we aimed to evaluate the effects of local intra-wound VP administration to decrease SSIs particularly in elderly patients with comorbidities after having undergone partial hip replacement in the treatment of intertrochanteric (ITF) or femoral neck fractures (FNF).

## Materials and methods

Our study was a retrospective clinical study performed according to the principles of the World Medical Association Declaration of Helsinki, “Ethical Principles for Medical Research Involving Human Subjects” (revised in 2013) [6]. After the approval of the local ethical committee, records of consecutive patients between January 2019 and December 2019 who underwent partial hip replacement in the treatment of ITF or FNF were retrospectively reviewed by using the databases of two institutions. Collected data included patients’ discharge summaries, surgical reports, follow-up data, laboratory data, and X-ray images.

**Figure 1:**
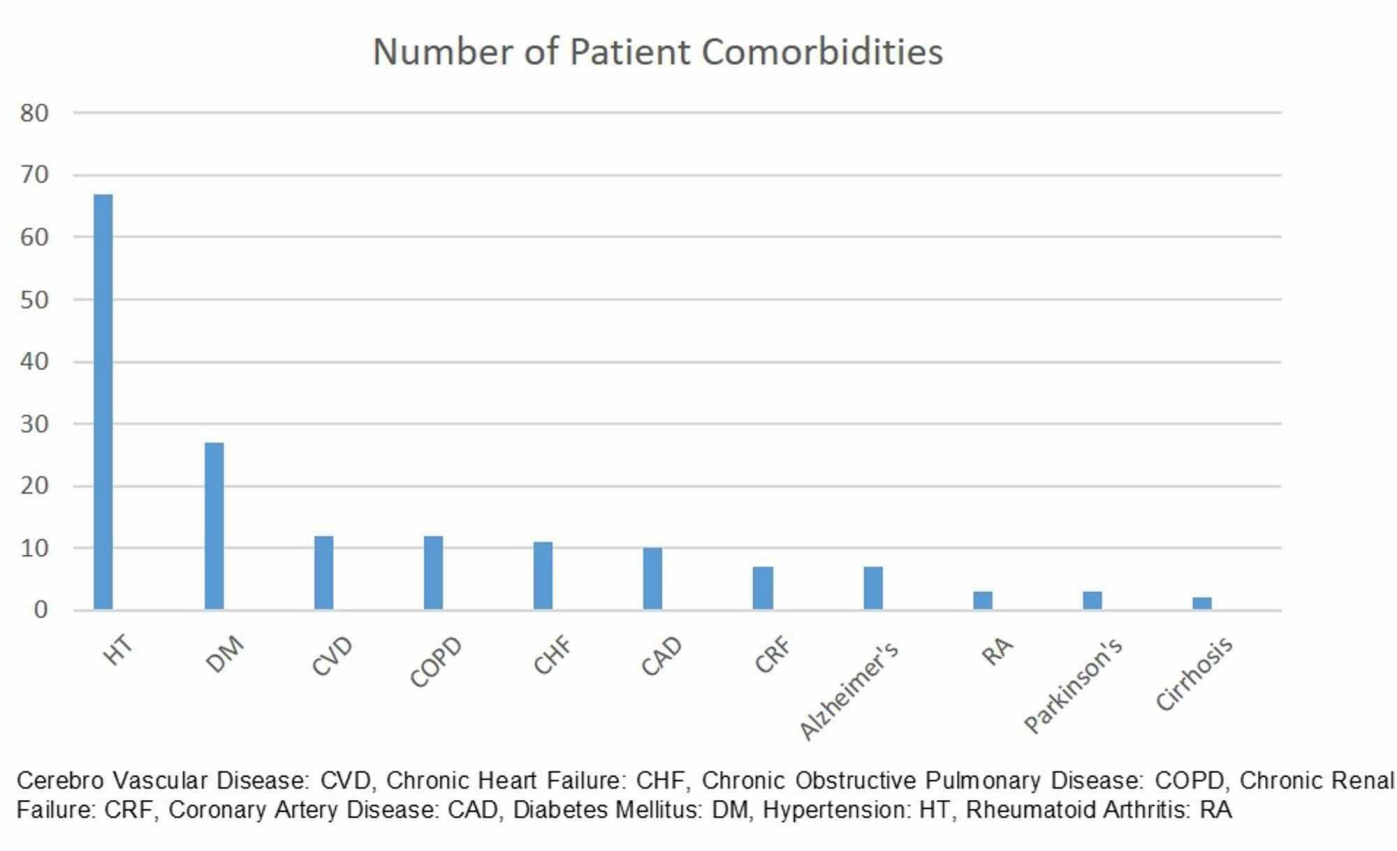
Number of patient comorbidities

We divided the patients into two groups. The control group (the non vancomycin-treated group) received standard systemic prophylaxis only, including 1 gr cefazolin IV (Cezol; Deva, Istanbul, Turkey) 30 minutes before the surgery, whereas the treatment (the vancomycin-treated group) received 1 gr of VP (Vancotek; Koçak Farma, Istanbul, Turkey) in the surgical wound just before surgical closure in addition to the systemic prophylaxis. We included patients of 64 years or older who had one or more comorbidities. We excluded patients with a pathological fracture from our study. We also excluded healthy individuals with no comorbidities from our study. Comorbidities of the patients included in our study are summarized in Figure 1.

We classified the fractures according to the AO classification system [7]. We included the patients AO type A1, A2, and A3 ITF and, AO type B1, B2, B3 type FNF in the study. We compared the post-operative SSI rates between the non vancomycin-treated group and the vancomycin-treated group.

Age, gender, fracture side, fracture type, days of hospitalization, patient comorbidities, whether or not local VP was applied in the surgical site, post-operative follow-up periods, post-operative SSIs, and post-operative intensive care unit (ICU) hospitalization of the patients were recorded. When applicable, complications that developed after surgeries were also recorded.

We analyzed the data using the IBM Statistical Package for Social Sciences (SPSS) software, version 22 (SPSS Inc, Chicago, IL, USA). Non-parametric tests were used for variables without normal distribution. Categorical data were compared with the chi-square test and Fischer’s exact test. Quantitative data were specified as mean, standard deviation, median, quarter scale, minimum and maximum values; p<0.05 was accepted as statistically significant.

## Results

A total of 93 patients (31 male and 62 female) met the inclusion criteria and were included in the study. The mean age of the patients was 81.8 (range: 64-94). The mean hospitalization time was 4.8 days (range 2-15) The fracture side of 50 patients was on the right side and 43 were on the left side. All patients had a history of a simple fall. All patients received the same pre-operative antibiotics protocol including 1 gr cefazolin IV 30 minutes before the surgical incision. During the surgery just before closing the wound, introperative local VP was administered to 35 of the 93 patients. There was a follow-up in the post-operative ICU in the early post-operative period for 32 of the patients. The mean time of their respective follow-up periods in the ICU was 1.3 days (range 1-3). The demographic data of the patients are summarized in Table 1.

**Table 1:** Demographic data of patients Age: Mean + SD (Standard deviation)

	Vancomycin Group		Non-Vancomycin Group	TOTAL
No of Patients	35 (%37.6)	58 (%62.4)		93
Sex				
Female	20 (%32.3)	42 (%67.7)		62 (%66.7)
Male	15 (%48.4)	16 (%51.6)		31(%33.3)
Age	81.88 + 7.54	81.87 + 6.57		82.88 + 6.91
Days of Hospitalization	5.6 + 2.4	4,4 + 1.53		4.9 + 1.98
No of Patients Followed in the ICU	11 (34.6%)	21 (65.6%)		32 (34.4%)
No of Patients with SSI	2 (33.3%)	4 (66.7%)		6 (6.5%)

Non-infectious complications included luxation in three patients and periprosthetic fracture in one patient. We detected post-operative wound infection in six of the 93 patients (6.4%). The bacterial cultures of the specimens taken from the wound discharges showed *Pseudomonas *species in three, *Acinetobacter baumanni* in two, and *Klebsiella aerogenes* in one patient, respectively. It was found that four of the patients who developed post-operative wound infection were in the non vancomycin-treated group and two were in the vancomycin-treated group.

The rate of the infection were found to be 5.7% in the vancomycin-treated and 6.9% in the non-vancomycin treated group and showed no statistically significant difference (p:0.498) (Table 2). Five of the six patients who developed post-operative infection had follow-ups in the ICU in the post-operative period. The incidence of SSI was statistically higher in the patients who had follow-ups in the post-operative ICU in the early post-operative period, than patients who were admitted to the ward without a follow-up in the ICU (p:0.017).

**Table 2:** Comparison of surgical site infection (SSI) between groups

	SSI -	SSI+	TOTAL	P value
Vancomycin group	33(94.3%)	2(5.7%)	35(37.6%)	0.498
Non-vancomycin group	54(93.1%)	4(6.9%)	58(62.4%)	
TOTAL	87(93.5%)	6(6.5%)	93	

When VP use and infection rates of 32 patients who had follow-ups in the ICU were examined specifically, it was seen that VP was not administered to 21 of these 32 patients (65.6%) and VP was administered to the remaining 11 of 32 (34.4%). SSIs were seen in two patients (18%) in the vancomycin group and three patients (14%) in the non-vancomycin group (Table 3). No statistically significant difference was found between the vancomycin and non-vancomycin groups in terms of infection rate in patients who had follow-ups in the ICU (p:0.363) (Table 3).

**Table 3:** Comparison of surgical site infection (SSI) between groups in patients that had follow-ups in the ICU

	SSI -	SSI+	TOTAL	P value
Vancomycin group	9(82%)	2(18%)	11(34.4%)	0.363
Non-vancomycin group	18(86%)	3(14%)	21(65.6%)	
TOTAL	27(85%)	5(15%)	32	

When we specifically searched VP administration and SSI rates in diabetic and non-diabetic patients, we detected SSIs in three of the 27 patients (11%) in the diabetic group and three of the 66 patients in the non-diabetic group (4.5%). This difference was not statistically significant (p:0.232). 19 of 27 patients in the diabetic group did not receive VP application, while eight of them received VP application. Two (10.5%) of the 19 diabetic patients in the non-vancomycin group had wound infection, while one (12.5%) of the eight diabetic patients in the vancomycin group developed infection. This difference was not statistically significant (p:0.669).

In the non-diabetic patient group, 27 of 66 patients had been administered VP. One (2.5%) of the 39 non-diabetic patients in the non-vancomycin group had SSI, while two (7.4%) of the 27 non-diabetic patients in the vancomycin group developed SSIs. This difference was not statistically significant (p:0.637).

## Discussion

Literature supports that the administration of VP in the surgical site reduces the risk of post-operative SSIs. In a study by O’Neill et al. [5], in which they retrospectively evaluated 110 patients, the authors concluded that the use of VP in surgical wounds may significantly reduce the incidence of SSI in patients with traumatic spine injuries treated with PSFI. Zebala et al. [4] assessed the efficacy of intrawound VP in eradicating bacterial surgical site contamination in a rabbit spine surgery model and showed that intrawound VP administration in combination with pre-operative IV cefazolin eliminated surgical site contamination caused by *Staphylococcus aureus*. All rabbits that were administered only prophylactic cefazolin had persistent *Staphylococcus aureus* contamination. Similarly, a recent study Karau et al. [8] showed that VP application prevented methicillin-resistant *Staphylococcus epidermidis* (MRSE) infection in a rat implant model. Karau et al. also showed that VP and vancomycin suspended within poly(lactic-co-glycolic acid) microspheres (MS) were active against MRSE in vitro [8]. However, vancomycin MS was found to be inferior to the topical VP. In a multicenter study, Horii et al. [9] examined if intrawound VP could prevent SSIs after spinal surgery with posterior instrumentation. After a retrospective review of 2859 patients, the authors performed a propensity score-matched analysis and concluded that intrawound application of VP was not associated with a significant decrease in the incidence of SSIs after spinal surgery with posterior instrumentation. However, in this study, the rate of infections caused by the *Staphylococcus* species was lower in the vancomycin group. Also, a systematic review and meta-analysis by Bakhsheshian et al. [10] examined the current clinical evidence on the use of VP in spine surgery and calculated the probability of developing a deep infection with intrawound VP as 0.23 times the probability of experiencing an infection without intrawound vancomycin. In that study for combined superficial and deep infections, the probability ratio was found to be 0.43. The authors concluded that clinical data supported the use of vancomycin to prevent SSIs in adult spine surgeries. Edelstein et al. [11] searched the efficacy of intra-articular VP in preventing infection in a rat model of a contaminated intra-articular implant. They inoculated the knee joint with methicillin-resistant *Staphylococcus aureus* (MRSA). As a result, they showed that the use of intra-articular VP in combination with systemic vancomycin completely eliminated the MRSA bacterial contamination. However, animals treated with systemic vancomycin alone had persistent MRSA contamination.

A single-center study [12] evaluated the microbiological patterns of post-operative SSIs after prophylactic use of VP in adult patients undergoing spinal deformity surgery. The authors retrospectively reviewed 1200 patients, where they administered crystalline VP to the surgical bed for infection prophylaxis. They had an SSI rate of 2.83%. 74% of the SSIs cultures were positive, with about half the organisms being gram-negative, such as *Citrobacter freundii*, *Proteus mirabilis*, *Morganella morgani*, and *Pseudomonas aeruginosa*. They concluded that in the setting of prophylactic topical VP use, the majority of SSIs are either caused by gram-negative organisms or are polymicrobial. Similarly, in our study, the SSIs are caused by gram-negative bacteria. The specimens taken from the wound discharges of our patients showed *Pseudomonas *species in three, *Acinetobacter baumanni* in two, and *Klebsiella aerogenes* in one patient, respectively. All of these organisms are defined as multidrug-resistant gram-negative bacteria and well-known pathogens responsible for nosocomial infections. These infections are most probably resistant to vancomycin and occur particularly in patients in ICUs [13,14]. The reason for the predominance of these bacteria might be due to the fact that five of the six patients in our study who developed post-operative SSI had follow-ups in the ICU in the early post-operative period.

Additionally, there are only a limited number of clinical studies in the literature that evaluates the effect of intrawound VP administration on reducing infection after hip arthroplasty. Omrani et al. [15] showed that local vancomycin administration reduced SSIs without any related complications in a study of 125 patients who underwent cemented total hip arthroplasty. In a recent retrospective study [16] which evaluated 555 patients who underwent cemented hip replacement, the authors applied intrawound local VP to 309 patients. Periprosthetic infection developed in two of the local vancomycin-treated group and four of the untreated group, respectively. They showed that absolute risk reduction was 0.98%, and 102 patients needed to be treated with topical vancomycin to prevent one periprosthetic joint infection. Similarly, Otte et al. [17] compared the rate of early prosthetic joint infections both with and without the use of intrawound VP during joint arthroplasty. They reported a significant reduction in the overall incidence of early post-operative infections following joint arthroplasty. However, only the revision procedures demonstrated a significant reduction in the rate of early post-operative infections in their study. Regarding the safety of the VP application for total joint arthroplasty, Johnson et al. [18] measured serum and wound concentrations of vancomycin after intrawound VP administration. They reported that VP reached highly therapeutic intrawound concentrations while yielding low systemic levels after total joint arthroplasty. Contrary to these previous reports supporting the use of local vancomycin, Hanada et al. [19] evaluated local vancomycin application after 166 consecutive patients that underwent primary total knee arthroplasty (TKA) or unicompartmental knee arthroplasty (UKA) and reported no significant decrease in the incidence of post-operative infection occurrence in primary TKA and a significant increase in aseptic wound complications after intrawound VP administration.

Another factor to consider when applying local vancomycin is its cost to the health system. In a cost-benefit analysis of local application of VP in posterior spinal fusion for spine trauma, Godil et al. [20] reported that the use of adjuvant VP was associated with a significant reduction in the incidence of post-operative infection as well as infection-related medical cost. They evaluated 110 patients retrospectively and found a 13% infection rate in the control group and 0% in the treatment group. Theologis et al. [21] also reported that local application of VP significantly decreased SSI for adults undergoing spinal reconstructive surgery with a savings in costs of $244,402 per 100 thoracolumbar adult deformity procedures. Similarly, cost-effective analysis in spine surgery, Hatch et al. [22] demonstrated the prophylactic administration of local VP during shoulder arthroplasty to be a highly cost-effective practice. However, to our current knowledge, there is no cost-benefit analysis evaluating SSIs after hip arthroplasty in the literature.

The major limitation of the present study is the limited number of patients and its retrospective nature. Future studies with a larger prospective randomized patient group comparing vancomycin-treated and non vancomycin-treated groups would be beneficial in better understanding the effects of VP administration to reduce the risk of SSI, particularly in elderly patients with comorbidities.

## Conclusions

In conclusion, our study on local application of VP in surgical wounds was found to be ineffective in reducing the incidence of SSI after partial hip replacement in elderly patients with comorbidities. Since elderly patients with comorbidities more frequently have follow-ups in the ICU in the post-operative period and are more prone to post-operative SSIs, particularly to nosocomial infections than healthy individuals, the use of local VP might not be as effective as its use in younger and healthier patients undergoing partial hip replacement.
